# Void Fraction Measurement of Oil–Gas–Water Three-Phase Flow Using Mutually Perpendicular Ultrasonic Sensor

**DOI:** 10.3390/s20020481

**Published:** 2020-01-15

**Authors:** Weikai Ren, An Zhao, Ningde Jin

**Affiliations:** School of Electrical and Information Engineering, Tianjin University, Tianjin 300072, China; renweikai1222@tju.edu.cn (W.R.);

**Keywords:** oil–gas–water flows, void fraction, mutually perpendicular ultrasonic sensor

## Abstract

The complex flow structure and interfacial effect in oil–gas–water three-phase flow have made the void fraction measurement a challenging problem. This paper reports on the void fraction measurement of oil–gas–water three-phase flow using a mutually perpendicular ultrasonic sensor (MPUS). Two pairs of ultrasonic probes are installed on the same pipe section to measure the void fraction. With the aid of the finite element method, we first optimize the emission frequency and geometry parameters of MPUS through examining its sensitivity field distribution. Afterward, the oil–gas–water three-phase flow experiment was carried out in a vertical upward pipe with a diameter of 20 mm to investigate the responses of MPUS. Then, the void fraction prediction models associated with flow patterns (bubble flow, slug flow, and churn flow) were established. Compared to the quick closing valves, MPUS obtained a favorable accuracy for void fraction measurement with absolute average percentage error equaling 8.983%, which indicates that MPUS can satisfactorily measure the void fraction of oil–gas–water three-phase flow.

## 1. Introduction

Oil–gas–water three-phase flow is frequently encountered in the exploitation of oil and natural gas. Determining the individual phase flow rate of oil–gas–water three-phase flow is of significant importance for optimizing the performance of oil well production and enhancing oil recovery. Due to the existence of two dispersed phases, the interfacial interaction between the phases becomes complex, and enhances the slippage effect. The investigation of highly random, irregular and unstable flow structural of oil–gas–water three-phase flow is difficult to conduct using computational fluid dynamics method, which poses a great challenge for void fraction measurement [1,2,3,4]. Traditional void fraction measurement is mostly based on conductance and capacitance. Although attractive due to its simplicity and effectiveness, the electrical methods is disabled to distinguish gas and oil phases for they are both non-conductive phases. Because gas and liquid with obvious density difference have well-recognized characteristics, the ultrasonic method is extended to void fraction measurement of oil–gas–water three-phase flow [5,6,7,8,9,10].

In earlier studies, the reason that restricts the application of ultrasonic method void fraction measurement is the deleterious effect of standing waves accompanied by a continuous wave ultrasonic sensor [7]. To address the problem, Xu et al. [6] employed a pair of pulsed ultrasonic transducers positioned opposite to each other to measure the void fraction of gas–liquid two-phase flow and found that the pulsed ultrasonic method not only can avoid standing waves, but also has a sensitive measurement for low gas concentrations. Passed through the fluid, the attenuation of ultrasonic pulse energy contains the information about void fraction. According to the reflection law of ultrasonic energy in a gas–liquid interface, the relationship between ultrasonic energy attenuation and gas holdup is established [11]. In addition to the physical void fraction prediction model, Carvalho et al. [12] designed a neural network for void fraction in oil–gas–water three-phase flow based on ultrasonic method which consisted of one emitter and three receivers at different positions where the signal is obtained to feed the neural network. Furthermore, ultrasonic method is employed to the bubble diameter measurement and the velocity measurement [13,14,15,16,17,18].

Focusing on void fraction measurement in oil–gas–water three-phase flow, because of the heterogeneous distribution of the gas phase. The ultrasonic sensor consisted of single receiver is insufficient to accurately measure void fraction [19,20]. In this paper, a mutually perpendicular ultrasonic sensor (MPUS) system composed of two pairs of ultrasonic transducers positioned opposite to each other was designed to measure the void fraction of heterogeneous flow in oil–gas–water three-phase flow. Firstly, the emission frequency and geometry parameters of MPUS was optimized, and a dynamic experiment was conducted to verify the reliability of the sensor. The result suggests that MPUS can accurately measure the void fraction of oil–gas–water three-phase flow.

## 2. Optimization of the Mutually Perpendicular Ultrasonic Sensor (MPUS)

**Figure 1:**
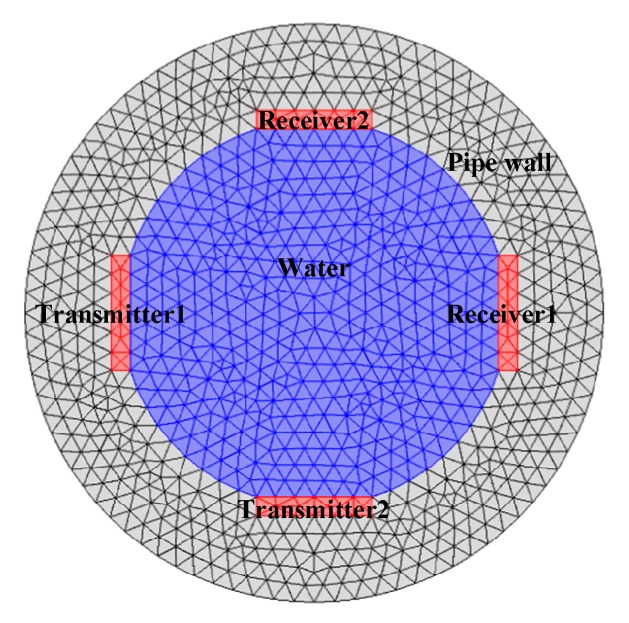
The two-dimensional meshed models of a mutually perpendicular ultrasonic sensor (MPUS).

In order to design the MPUS applicable for void fraction measurement of oil–gas–water three-phase flow, the optimization of the ultrasonic probe diameter and ultrasonic pulse frequency were implemented in this part. The multi-physical field coupling simulation software COMSOL was used to establish the two-dimensional meshed models of MPUS as shown in Figure 1. The simulation was conducted by element number of 85224. The continuous wave equation of ultrasonic wave in the frequency domain follows the following equation:

(1) $$\nabla \cdot \left(-\frac{1}{\rho_{s}}\left(\nabla p\right)\right)-\frac{\omega^{2}p}{\rho_{s}c_{s}^{2}}=0$$

where *p* is the sound pressure, *ρ_s_* is the medium density, ω is the acoustic angular frequency, $c_{s}$ is the sound velocity.

Two index termed average sensitivity *S_avg_* and homogeneous error *SVP* was introduced to give a quantitative optimization. The sensitivity definition is as follows: first, the sound pressure levels (sound pressure level is the logarithmic representation of the effective value of sound pressure) of two receivers when the pipe was full of water were calculated as *SPL*_1_(*w*) and *SPL*_2_(*w*), respectively. The purpose of the simulation was to investigate the sensitivity of the sensor to gas, and in the oil–gas–water three-phase flow oil and water were mixed into an emulsified state and it was difficult to simulate the mixture of oil and water. So the fluid in the simulation did not mix oil. Then *SPL*_1_(*g*) and *SPL*_2_(*g*) were calculated when the pipe is full of gas. A bubble with the diameter of 1 mm at (*x*,*y*) was put in the pipe full of water, the bubble in the oil–gas–water three-phase flow had several diameters, but the measurement of small bubbles is an important factor to limit the sensitivity of ultrasonic sensors [19]. Based on our previous research, the smallest bubble was 1 mm in diameter [21]. The sound pressure level of two receiver is *SPL*_1_(*x*,*y*) and *SPL*_2_(*x*,*y*). The sensitivity *S*(*x*,*y*) of MPUS can be defined as:

(2) $$S_{i}\left(x,y\right)=\frac{SPL_{i}\left(w\right)-SPL_{i}\left(x,y\right)}{SPL_{i}\left(w\right)-SPL_{i}\left(g\right)}\;(i=1,2)$$

(3) $$S\left(x,y\right)=\frac{1}{2}\left[S_{1}\left(x,y\right)+S_{2}\left(x,y\right)\right]$$

Changing the bubble (*x*,*y*) to different locations on the section of the pipe, the sensitivity at every position can be obtained. We defined the average sensitivity *S_avg_* of the MPUS as follows:

(4) $$S_{avg}=\frac{1}{M}\sum_{j=1}^{M}S_{j}$$

where *S_j_* represents the sensitivity at the *j* test position, and *M* is the total number of all positions. Meanwhile, the homogeneous error *SVP* is defined:

(5) $$SVP=\frac{S_{dev}}{S_{avg}}$$

(6) $$S_{dev}={\left(\frac{1}{M}\sum_{j=1}^{M}{\left(S_{j}-S_{avg}\right)}^{2}\right)}^{1/2}$$

**Figure 2:**
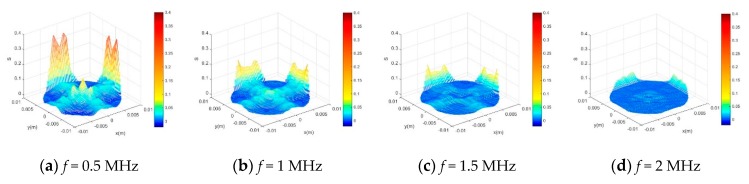
Sensitivity distribution of the MPUS under different ultrasonic pulse frequency.

We used single factor alternate method to optimize the ultrasonic probe diameter *D* and ultrasonic pulse frequency *f*. First, when the diameter of the ultrasonic probe was fixed as *D* = 6mm, the ultrasonic pulse frequency was changed to 0.5 MHz, 1 MHz, 1.5 MHz and 2 MHz respectively, to calculate the acoustic field sensitivity distribution as shown in Figure 2. *S_avg_* and *SVP* which were extracted from the sensitivity field shown in Figure 2, and the result is shown in Table 1. We find that MPUS has high *S_avg_* and minimum *SVP* when ultrasonic pulse frequency *f* = 1 MHz, so the ultrasonic pulse frequency *f* of MPUS was determined to be 1 MHz.

**Figure 3:**
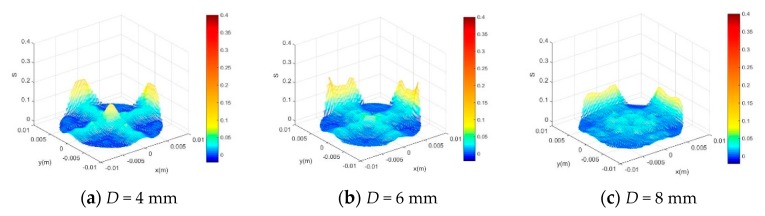
Sensitivity distribution of the MPUS under different ultrasonic probe diameter.

After determining the optimized ultrasonic pulse frequency of 1 MHz, we changed the ultrasonic probe diameter to investigate the influence on the sensitivity distribution. Figure 3 shows the sensitivity distribution when the ultrasonic pulse frequency was fixed at 1 MHz and the probe diameter *D* was 4 mm, 6 mm and 8 mm respectively. *S_avg_* and *SVP* were also extracted from the sensitivity field as shown in Table 2. The result suggests that MPUS has the highest *S_avg_* and low *SVP* when *D* = 6 mm, so the ultrasonic probe diameter of MPUS was determined to be 6 mm.

## 3. Experimental Test

**Figure 4:**
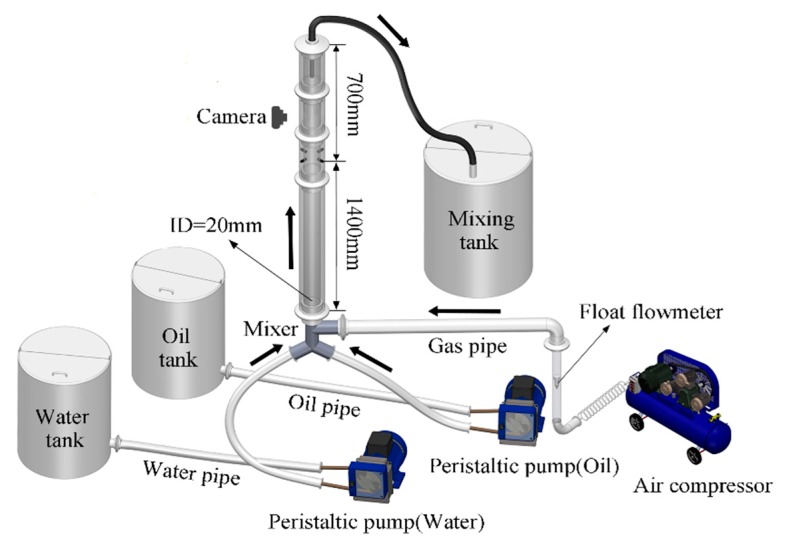
The sketch map of oil–gas–water three-phase flow experimental facility.

The experiment of oil–gas–water three-phase flow was conducted in multiphase flow sensor system and fluid flow laboratory in Tianjin University (Tianjin, China). The sketch map of experimental facility is shown in Figure 4. It includes a water tank, oil tank, mixed tank, air compressor, gas flowmeter, two industrial peristaltic pumps (Lead Fluid, WG600F, accuracy: ±0.2%, range: 0.822–2879 mL/min) were used to transport and meter tap water and 3# industrial white oil with the density of 807 kg/m^3^ and viscosity of 3.5 mPa·s from the respective tank. The air compressor was used to generate the required gas for the experiment which was measured by the gas flowmeter and then entered the pipeline. The vertical upward testing pipe was an acrylic tube with inner diameter of 20 mm and outer diameter of 25 mm. After flowing through the measurement section, mixed fluid was drained into a 300-L mixing tank for automatic separation by gravity. A high-speed camera (Photron, FastCam Mini UX50/100) was installed to capture the flow state of the mixed fluid, and the interval of each frame was 0.0075 s. The MPUS was 1400 mm away from the pipeline inlet.

Firstly, the gas flow rate *Q_g_* and oil cut *f*_o_ of liquid phase *f*_o_ was fixed. We obtained the multichannel output signal from MPUS in different flow conditions with the gradual increase of oil–water mixed liquid flow rate *Q_l_*. Next we change *f*_o_ to conduct experiment. Finally, we changed *Q_g_* and repeated the above experimental procedure. During the entire process, *Q_g_* increased from 1.5 m^3^/day to 12 m^3^/day, *Q_l_* changes from 1 m^3^/day to 32 m^3^/day, and *f*_o_ ranged from 0% to 20%.

**Figure 5:**
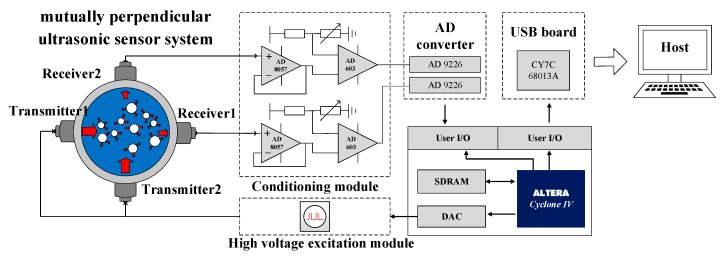
Sketch map of mutually perpendicular ultrasonic sensor system.

The MPUS system is shown in Figure 5. It was composed of two pairs of ultrasonic transducers positioned opposite to each other, high voltage excitation module, conditioning module, AD converter, USB board and FPGA. Under the control of FPGA, two transmitters were excited to generate ultrasonic pulses by a high-voltage electrical pulse signal with amplitude of 100 V and frequency of 1 KHz generated from the high voltage excitation module. Ultrasonic pulses were obtained by receivers after through the mixed fluid.

**Figure 6:**
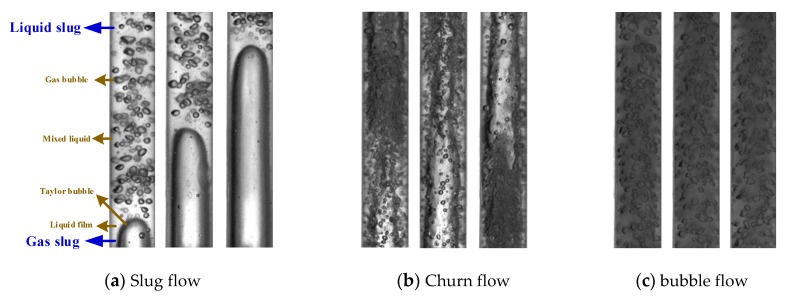
Snapshots of typical flow patterns of oil–gas–water three-phase flow captured by camera.

Direct observation through a high-speed camera is the most effective method to identify the flow pattern. When the liquid flow rate is low, the flow pattern presents as slug flow (*Q_g_* = 1.5 m^3^/day, *Q_l_* = 1.0 m^3^/day, *f*_o_ = 0.02). Figure 6a shows the fluid structure of slug flow taken by high-speed camera. A pseudo-periodic rise of Taylor bubbles is separated by liquid slugs that include dispersed gas bubbles between the Taylor bubble and the pipe wall. With the liquid flow rate increasing (*Q_g_* = 12 m^3^/day, *Q_l_* = 20 m^3^/day, *f*_o_ = 0.02), the flow pattern gradually transits to the churn flow as shown in Figure 6b. Impacted by the liquid slug, the Taylor bubble gradually distorts and deforms. When the gas flow rate is low and the liquid flow rate is high (*Q_g_* = 12 m^3^/day, *Q_l_* = 20 m^3^/day, *f*_o_ = 0.02), the gas phase is crushed into small bubbles, which are randomly distributed in the fluid as shown in Figure 6c.

## 4. Results and Discussion

**Figure 7:**
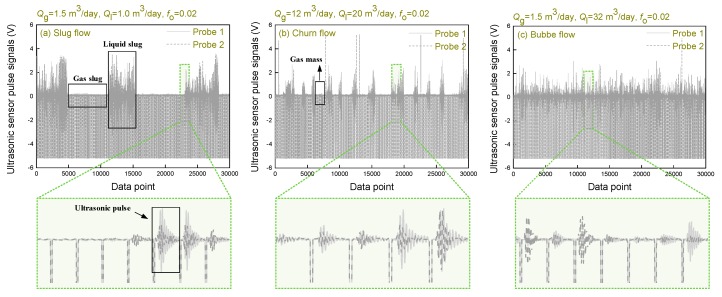
Ultrasonic pulse signals of typical flow patterns of oil–gas–water three-phase flow.

The ultrasonic pulse signals from two receivers under typical flow patterns is shown in Figure 7. The difference between the signals received by the two receivers is obvious, which confirms the heterogeneous structure of the oil–gas–water three-phase flow. When the flow pattern presents slug flow, a pseudo-periodic rise of Taylor bubbles separated by liquid slugs that include dispersed gas bubbles, the corresponding ultrasonic pulse signals is also periodic as shown in Figure 7a. When the ultrasonic pulse hits the gas slug, all the energy is reflected, and the receiver barely receives the ultrasonic pulse. When the ultrasonic pulse encounters the liquid slug, part of the energy is attenuated due to scattering effect, and the remaining energy is received by the receiver. The received energy is related to the void fraction in the liquid slug. In the churn flow, there is some gas mass which can reflect the ultrasonic pulse. So the ultrasonic pulse signals of churn flow also present periodicity. By contrast with slug flow, the ultrasonic pulse signals correspond to shorter duration than the gas slug, and the signals corresponding to the liquid slug of the churn flow have lower amplitude than that of the slug flow. When the flow pattern presents bubble flow, all the ultrasonic pulse can arrive at the receivers, and the received energy is related to the void fraction.

**Figure 8:**
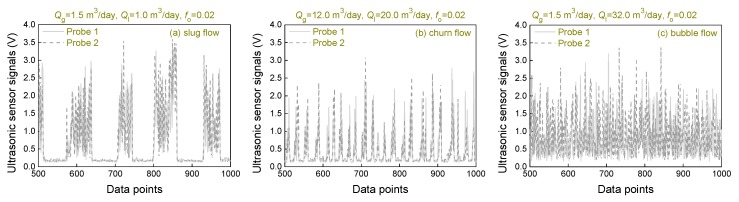
Ultrasonic pulse maximum amplitude sequence of oil–gas–water three-phase flow.

In order to quantify the energy of each ultrasonic pulse and obtain the void fraction, we extracted the maximum amplitude value of each ultrasonic pulse, and the results are shown in Figure 8.

The propagation attenuation law of ultrasonic wave in medium can be expressed as:

(7) $$I/I_{0}=\exp\left(-\alpha_{0}L\right)$$

where *I*_0_ is the transmitted energy, *I* is the measured incident energy when the path length is *L*, $\alpha_{0}$ is the attenuation coefficient. Since the energy of the mechanical wave is proportional to the square of the amplitude, the above equation can be converted into:

(8) $$A/A_{0}=\exp\left(-\alpha L\right)$$

where *A*_0_ is the maximum amplitude of ultrasonic pulse when only liquid phase is measured, *A* is the maximum amplitude of ultrasonic pulse when there are bubbles in the medium, α is the absorption coefficient. The above equation is valid on the premise that the gas phase is uniformly distributed in the medium. Therefore, Equation (8) can be only used to measure the void fraction of bubble flow. As for slug flow and churn flow, we will give the specific measurement scheme below.

We used the ultrasonic sensor to measure oil–water two-phase flow in a previous work, and the results suggested that the attenuation of ultrasonic pulse is not significant when the diameter of the oil bubble is less than 1 mm [20]. In the oil–gas–water three-phase flow, due to the injection of gas, oil and water have been mixed into an emulsified state. At this time, the oil droplets are about 0.2 mm in diameter [22]. On the other hand, compared to gas bubbles with a huge density difference, the loss of energy at the gas–liquid interface is much greater than that at the oil–water interface. Therefore in oil–gas–water three-phase flow, we can assume that the maximum amplitude of ultrasonic pulse when only liquid phase is measured can be obtained by:

(9) $$A_{0}=f_{w}A_{w}+(1-f_{w})A_{o}$$

where *A_w_* is the maximum amplitude of ultrasonic pulse when only water is measured, *A_o_* is the maximum amplitude of ultrasonic pulse when only oil is measured, *f_w_* (*f_w_* = 1 − *f*_o_) is the water holdup. Stravs et al. [11] developed Equation (8):

(10) $$A/A_{0}=-\exp\left(\frac{aL}{8\theta }\cdot \frac{nd_{sm}}{2}\right)$$

where *a* is the volumetric interfacial area and *θ* is the scattering coefficient which is a constant; *n* = 2π/λ represents the wave number of the ultrasonic waves, *λ* is the ultrasound wavelength, and *d_sm_* is the Sauter mean bubble diameter. Sauter mean diameter *d_sm_* can be related to void fraction *Y_g_*:

(11) $$a=\frac{6Y_{g}}{d_{sm}}$$

Substituting Equation (10) into Equation (11), we can obtain the void fraction prediction model for oil–gas–water three-phase bubble flow in terms of maximum amplitude of ultrasonic pulse signal:

(12) $$-\ln\left(A/A_{0}\right)=\left(\frac{3n}{8\theta }\right)Y_{g}L$$

As for slug flow and churn flow, fluid structure can be divided into gas slug and liquid slug in two parts, and then the void fraction can be expressed as:

(13) $$Y_{g}^{*}=w_{g}\cdot Y_{g,gas}+w_{l}\cdot Y_{g,liquid}$$

where *Y_g,gas_* is the void fraction of gas slug, *Y_g,liquid_* is the void fraction of liquid slug, *w_g_* and *w_l_* is the proportion of gas slug and liquid slug. The Taylor bubble almost filled the pipe, so *Y_g,gas_* equals 1 approximately. The method ignores the liquid film around the Taylor bubble, which causes the error. On the other hand, the liquid slug has a similar structure to bubble flow, and we can get the *Y_g,liquid_* according to Equation (12).

**Figure 9:**
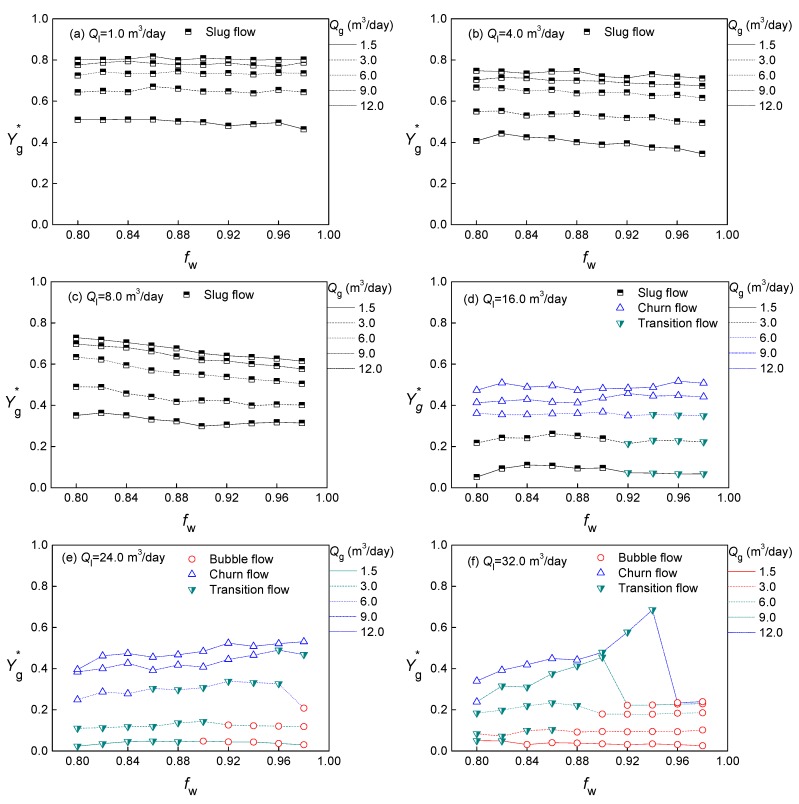
The void fraction predicted results of different flow conditions.

Through the above analysis, the prediction models for different flow patterns of gas–oil–water three-phase flow are established. The void fraction predicted results $Y_{g}^{*}$ of different flow conditions are shown in the Figure 9. It is obvious that the water cut *f_w_* has little influence on the void fraction when the fluid structure is stable. Because of the Taylor bubble, the void fraction of the slug flow is the highest. The bubble flow occurs when the liquid flow rate is high, so the void fraction is the lowest and does not exceed 0.3. The void fraction of the churn flow with unstable structure has a wide range, and is susceptible to *f_w_*.

**Figure 10:**
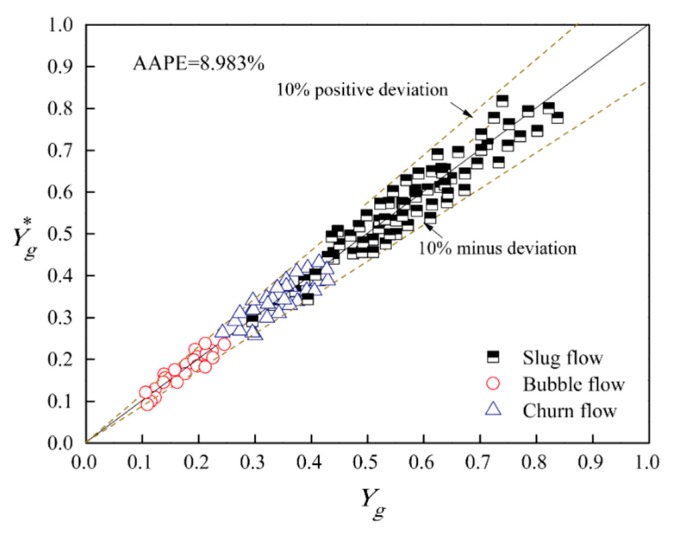
The void fraction predicted results of MPUS.

For assessing the measurement accuracy of the void fraction of oil–gas–water three-phase flow using MPUS, we compared the void fraction predicted results $Y_{g}^{*}$ with the void fraction $Y_{g}$ obtained by quick closing valves (QCVs). Using QCVs is a well-tested method to assess the accuracy of measurement of flow parameters of multiphase flow. Therefore in this paper, we adopted a pair of QCVs to measure the void fraction as the comparison. For bubble flow, QCVs can accurately obtain the void fraction of oil–gas–water three-phase flow. For slug and churn flow, we conducted the optimal design for QCVs in a previous study [23]. In order to be accurate, the void fraction of each flow condition was measured five times by the QCVs, and the average value was regarded as the true void fraction. The absolute average percentage error (AAPE) is utilized to quantitative the comparison result in Figure 10. The result suggests that MPUS obtains a favorable accuracy for void fraction measurement with AAPE equaling 8.983%.

## 5. Conclusions

In this study, an ultrasonic method was introduced to measure the void fraction of oil–gas–water three-phase flow. Firstly the optimization ultrasonic sensor system we termed the mutually perpendicular ultrasonic sensor was conducted using COMSOL. The diameter and frequency of ultrasonic probes were determined. In the experimental process, the MPUS was utilized to measure the void fraction of different flow patterns, and the corresponding prediction models of void fraction were established. Our conclusions can be summarized as follows.

1. Oil–gas–water three-phase flow has a complex flow structure and interfacial effect, and conventional sensors with a single direction are insufficient for accurate measurement of void fraction. MPUS can reduce the error caused by fluid inhomogeneity by setting two pairs of ultrasonic transducers positioned opposite to each other.
2. The prediction model of void fraction in the bubble flow can be established by the relationship between ultrasonic pulse attenuation and void fraction. Further based on the prediction model of bubble flow, the prediction models of the slug flow and churn flow were established through calculating the void fraction of gas slug and liquid slug respectively.
3. The measurement accuracy was assessed by comparing the void fraction predicted results with the void fraction obtained by quickly closing valves. The method ignored the liquid film around the Taylor bubble, which causes the error. MPUS can satisfactorily measure the void fraction of oil–gas–water three-phase flow with the final absolute average percentage error equaling 8.983%.

## Figures and Tables

**Table 1:** Optimized result of ultrasonic pulse frequency.

*f* (MHz)	*S_avg_*	*SVP*
0.5	0.02577	2.31425
1	0.0181	1.70519
1.5	0.01165	1.95735
2	0.00581	1.99357

**Table 2:** Optimized result of ultrasonic probe diameter.

*D* (mm)	*S_avg_*	*SVP*
4	0.01645	2.31425
6	0.0181	1.70519
8	0.01714	1.32984
